# Reliability of a semi-tethered front crawl sprint performance test in adolescent swimmers

**DOI:** 10.3389/fphys.2023.1260346

**Published:** 2023-12-13

**Authors:** Stefan Szczepan, Zofia Wróblewska, Sebastian Klich, Kamil Michalik, Tomohiro Gonjo, Bjørn Harald Olstad, Marek Rejman

**Affiliations:** ^1^ Department of Swimming, Faculty of Physical Education and Sport, Wroclaw University of Health and Sport Sciences, Wroclaw, Poland; ^2^ Faculty of Pure and Applied Mathematics, Wroclaw University of Science and Technology, Wroclaw, Poland; ^3^ Department of Paralympic Sport, Faculty of Physical Education and Sport Science, Wroclaw University of Health and Sport Sciences, Wroclaw, Poland; ^4^ Department of Human Motor Skills, Faculty of Physical Education and Sport Sciences, Wroclaw University of Health and Sport Sciences, Wroclaw, Poland; ^5^ Institute for Life and Earth Sciences, School of Energy, Geoscience, Infrastructure and Society, Heriot-Watt University, Edinburgh, United Kingdom; ^6^ Department of Physical Performance, Norwegian School of Sport Sciences, Oslo, Norway

**Keywords:** swimming, anaerobic capacity, semi-tethered swimming, sport-specific tests, adolescent swimmers

## Abstract

This study aimed to evaluate the test-retest reliability of a sprint performance test with semi-tethered front crawl swimming to indirectly assess the current potential to perform at maximal anaerobic effort in adolescent swimmers. Eight adolescent swimmers participated in this study (gender: females (n = 4) aged 13.0 ± 0.8 years, body height 1.6 ± 0.0 m, body mass 50.1 ± 4.5 kg; and males (n = 4) aged 13.3 ± 1.3 years, body height 1.7 ± 0.1 m, body mass 59.0 ± 8.2 kg. The testing protocol consisted of two trials of 25 m semi-tethered front crawl swimming with maximal effort and with 1 kg resisted isotonic load. Velocity data were recorded automatically by the 1080 Sprint device for 15 m (between 3 m and 18 m). The Fast Fourier Transform algorithm filtered raw instantaneous swimming velocity data in distance (time) function. A third-degree polynomial was used to extract the individual velocity profile, from which the following variables were chosen for test-retest reliability and the assessment of sprint performance: t_trial15_, v_max_, v_min_, tv_to max,_ tv_at max_, D_to_ v_max_, D_at_ v_max_, fatigue index. Parameters such as v_max_, v_min_, and t_trial15_ were estimated from swimming velocity profiles and considered as reliable. The CV showed low variance <5%; while ICC_2,1_ demonstrated respectively good (ICC_2,1_: 0.88), very good (ICC_2,1_: 0.95), and excellent (ICC_2,1_: 0.98) rate of relative reliability; and the Bland-Altman index revealed an acceptable agreement (LoA ≤5%) between two measurements. The sprint performance test based on semi-tethered front crawl swimming confirmed that t_trial15_, v_max,_ and v_min_ were reliable variables to indirectly indicate a potential to perform the maximal anaerobic effort among adolescent swimmers. The evaluation of the swimming velocity profiles allows coaches to monitor the adaptive changes of performance during the training process.

## Introduction

The primary assessment of anaerobic performance on land is based on the Wingate Anaerobic Test (WAnT) (Bar-Or, 1987), in which the obtained power of exercise is presented as a function of the trial time or covered distance. The maximal power level and the time to reach and maintain the velocity peak may indicate phosphagen capacity and the average power determines glycolytic capacity (Papoti et al., 2013). A previous study reported a strong correlation (r = 0.83) between maximal power measured on the cyclo-ergometer during WAnT and freestyle swimming time for 50 m (Hawley and Williams, 1991). The relationship between the time needed to swim 50 m of freestyle and mean power in WAnT performed with upper limbs (r = 0.63) and lower limbs (r = 0.76) were established by Hawley et al. (1992). Duche et al. (1993) found a strong correlation between the time necessary to swim 50 m of freestyle and the average power obtained in the WAnT test (r = 0.68). However, it is known that the specificity of the water environment, i.e., water density, buoyant force, or hydrodynamic drag forces (Truijens and Toussaint, 2005) largely influences aquatic exercise (Kimura et al., 1990). Thus, the diagnostic value of WAnT during land-based conditions is questionable for assessing the exercise capacity of swimmers because it does not reflect the actual free-swimming conditions (Smolka and Ochmann, 2013; Soares et al., 2014).

The evaluation of anaerobic performance in swimmers was also conducted using in-water tests. Shionoya et al. (1999), measured the net mechanical power calculated from the product of tethered force and swimming velocity in real-time (during swimming) with an ergometer located on land. Smolka and Ochmann (2013), used nonchronometric approaches during in-water tests similar to the WAnT. They analyzed a series of instantaneous swimming velocities over 100 m based on data recorded with a video camera. Bátorová et al. (2021) and Demarie et al. (2019), analyzed anaerobic performance using Inertial Measurement Units (IMU). Fernandes et al. (2008) and Neiva et al. (2011), introduced anaerobic critical velocity (AnCV), as a parameter to evaluate and monitor anaerobic training among swimmers.

The fully-tethered swimming method was also used to assess the anaerobic performance of swimmers in the water through a 30 s maximal front crawl swimming test (Nagle Zera et al., 2021). The swimmer does not move forward in fully-tethered swimming, and the propulsive movements generate zero horizontal velocity (Samson et al., 2019). Kjendlie and Thorsvald (2006) showed that a fully-tethered swimming power test was highly reliable (Cronbach’s *α* = 0.99). However, Samson et al. (2019) reported that the hand’s orientation is perpendicular to the surface of the water earlier than in free swimming, and velocity and acceleration are different in fully-tethered vs free swimming. Furthermore, fully-tethered swimming sustains the strength potential of a swimmer rather than the ability to apply force effectively (Ruiz- Navarro et al., 2020), leading to the overestimation of force (Santos et al., 2023). This gives rise to the question of underestimating the measurement of the kinematic of swimming stroke (e.g., acceleration and maximal velocity) which are significant predictors of an adolescent swimmer’s performance (Sokołowski et al., 2022) and consequently may result in an unrealistic view of the bioenergy of exercise (Thompson et al., 2004).

Another method helpful in evaluation of swimming performance is semi-tethered swimming. According to this approach, a swimmer moves forward in the water subjected to an external load, while the forward motion induces relative streamwise water flows around the body. Moreover, the swimmer maintains more natural swimming mechanics. It makes the semi-tethered swimming test more specific to the free-swimming condition compared to fully-tethered swimming (Hancock et al., 2015) depending on the load (Cuenca-Fernández et al., 2020). The semi-tethered method has been used to evaluate the net power output which was calculated by multiplying the speed and force data produced against an external load (Dominguez-Castells et al., 2013; Kimura et al., 2013). However, the athletes often swim with almost a constant velocity that makes the sum of the mean propulsive and resistive forces during one stroke cycle nearly zero. Hence, the power calculated in semi-tethered swimming considers the power against the external load and not the propulsive power produced by the swimmer (Gonjo et al., 2021). The different use of semi-tethered swimming might be useful to measure the velocity with different external loads to examine the interaction between load and velocity (Gonjo et al., 2020; Olstad et al., 2020; Gonjo et al., 2021). The maximum velocity at zero load corresponds to maximal velocity during free-swimming. Moreover, the force exerted by the swimmer at fully tethered swimming should match the magnitude of the tethered force produced by the maximum load at zero velocity. The semi-tethered swimming is a reliable methodology and can potentially be used to assess strength and velocity capabilities during swimming (Olstad et al., 2020).

Short time and maximum intensity of exercise are the main criteria for the rate of anaerobic pathway contributions, such as split into phosphocreatine and anaerobic glycolysis (Driss and Vandewalle, 2013). The activation of phosphagen energy sources contribute mainly to the velocity increase for the approximately first 3 seconds of exercise (Hirvonen et al., 1987). The sum of the time to reach v_max_ (tv_to max_ [s]) and maintain (tv_at max_ [s]) maximal velocity is included in the time of maximal phosphagen transformations (Hawley, 1995). During longer swimming sprints, the maximal velocity (v_max_) and minimum velocity (v_min_) were strong determinants of the time obtained in 100 m front crawl swimming (r = −0.90, r = −0.92, respectively) (Smolka and Ochmann, 2013). These variables related to anaerobic metabolism (phosphagen and glycolytic) show a significant relationship with swimming performance at distances of 50 m and 100 m (Vitor Fde and Böhme, 2010; Neiva et al., 2011). Furthermore, after only a few seconds of exhaustive work, the power produced by the ATP-PCr system decays, so that after 6 seconds it provides only half of the total energy requirements (Hawley, 1995). After 6–10 s of maximal exercise, the contribution of anaerobic energy from PCr and anaerobic glycolysis is essentially equivalent (Hargreaves and Spriet, 2020). Hence, the observation of free-swimming velocity patterns and fatigue over time during a maximal effort could provide information about a swimmer’s anaerobic potential and the dynamics of the ATP-PCr cycle to lactic acid transition (Smolka and Ochmann, 2013; Soares et al., 2014). The presented arguments allow for the assumption that a semi-tethered swimming test can be used as an objective tool for indirectly measuring a swimmer’s potential to perform at maximal anaerobic effort. The potential to perform maximal effort is defined as the work capacity (anaerobic) lasting approximately 15 s (Driss and Vandewalle, 2013).

Adolescents have unique physiological features that greatly differ from those of adults, including a lower ventilatory efficiency during progressive exercise (Cooper et al., 1987) and a lower anaerobic peak power (Van Praagh and Doré, 2002). Moreover, anaerobic capacity is known to increase during puberty so the relative anaerobic contribution to short-distance swims would likely be lower in prepubertal *versus* adult swimmers (Mezzaroba and Machado, 2014). Hence, adolescents have a lower work capacity and therefore are less efficient than adults (Ratel and Blazevich, 2017). In this cohort, anaerobic metabolism is in its developmental phase (Olbrecht, 2000), which increases the susceptibility to fatigue (Zając et al., 2023). Additionally, in adolescents, inexperienced swimmers, fatigue reactions are also a function of deficits in the quality of swimming technique (Bassan et al., 2016). Different swimming velocity profiles were also linked to the age and competitive level (Smolka and Ochmann, 2013; Soares et al., 2014; Nagle Zera et al., 2021). Consequently, a different swimming velocity profile is to be expected in adolescent swimmers than in adult swimmers.

Most investigations in the area of an indirect assessment of anaerobic performance in swimming concern adult swimmers (Soares et al., 2014; Nagle Zera et al., 2021; Ruiz-Navarro et al., 2022). Few studies exist which include adolescent subjects (Zamparo et al., 2000; Strzala and Tyka, 2009; Mezzaroba and Machado, 2014; Dekerle, 2021). There is a lack of research that has used field-based swim tests to examine the swimming velocity profiles in order to objectively monitor the current state of performing maximal anaerobic effort among adolescent swimmers, which is a unique feature of this study. Furthermore, based on a literature review, the current state of knowledge regarding the interpretation of the diagnostic value of various methods for assessing the anaerobic potential of swimmers show discrepancies. Moreover, there is a small number of scientific reports that determine the reliability of using a motorized resistance device with adolescent swimmers.

Therefore, the current study aimed to evaluate the reliability of a sprint performance test determining an individual profile of instantaneous swimming velocity from semi-tethered front crawl swimming to indirectly assess an adolescent swimmer’s current potential to perform the maximal anaerobic effort. It was hypothesized that the sprint performance test with semi-tethered front crawl swimming is a reliable method to indirectly indicate a potential to perform the maximal anaerobic effort among adolescent swimmers.

## Materials and methods

### Participants

Eight healthy, adolescent swimmers participated in this study. According to Curtis (2015), the chronological definition of adolescence includes the ages of 10–18. Four females aged 13.0 ± 0.8 years, body height 1.6 ± 0.0 m, body mass 50.1 ± 4.5 kg, and BMI 20.0 ± 2.0 kg m^-2^ and four males age 13.3 ± 1.3 years, body height 1.7 ± 0.1 m, body mass 59.0 ± 8.2 kg, and BMI 19.6 ± 0.6 kg·m^-2^. The swimmers had at least 3 years of experience in competitive swimming and trained five to eight times per week. Their 50 m front crawl performance (females 32.6 ± 2.3 s; males 28.6 ± 2.6 s) categorized them as “well trained” in their age group. No swimmer suffered from any illness or any other restrictions that could hinder their performance during the experiment. All participants were instructed to avoid strenuous physical exercise over the 24 h before data acquisition and were required to maintain their normal lifestyle and diet. The legal guardian and swimmers were given a detailed verbal and written explanation of the investigation’s aims, procedures, and any risks involved. The legal guardian of each athlete provided written informed consent prior to participation in the study. The local ethics committee approved the study design (reference number 47). All procedures adhered to the prerogatives set out in the Declaration of Helsinki.

### Testing protocol

**FIGURE 1:**
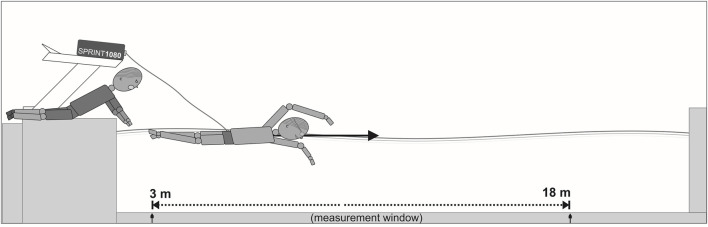
The test set-up for semi-tethered swimming trials.

The experiment was conducted in a short course indoor 25 m swimming pool (water temperature 27°C, air temperature 28°C, and relative humidity 60%). The 1080 Sprint (1080 Motion, Lidingö, Sweden) was used for data acquisition in semi-tethered swimming (Figure 1). The measurements were conducted in "isotonic” mode, meaning that the load was constant and independent of acceleration and deceleration throughout the entire experiment.

A 1.5 kW servo motor (2000 RPM OMRON G5 Series Motor, OMRON Corporation, Kyoto, Japan) provided stable resistance over the measurement window (Mangine et al., 2018). A composite fiber cord was attached to the motor and wrapped around a spool, extending up to 90 m. Participants wore a S11875BLTa swim belt (NZ Manufacturing, OH, United States) around their waist to connect the composite fiber cord. The resistance was controlled by the 1080 motion software app (1080 Motion, Lidingö, Sweden), which also recorded all kinetic data of the sprint trials with a frequency of 333 Hz. The mean error of the 1080 Sprint has previously been examined on the land condition and shown to be low across all measurements (velocity error ±0.5%, distance error = ± 5 mm, force error ±4.8 N) (Bergkvist et al., 2015). The device was placed on a starting block and secured with straps (1.0 m above the water level) to prevent the line from disturbing the lower limb movements of the swimmer (Amaro et al., 2017). Since the device was not placed at the water level, the horizontal velocity was calculated using the trigonometry function according to the to the following formula (1) Gonjo et al. (2021):

$$v=v_{\mathrm{abs}}\cdot \cos\left[\sin^{‐1}\left(1.0\cdot L_{c}^{‐1}\right)\right]$$ (1)

where: v is the horizontal component of the velocity data, v_abs_ is the absolute value of velocity from the software, 1.0 is the height (m) of the device from the water level (the point where the cord is stretched from the equipment), and L_c_ is the length of the wire (m) between the machine and the swimmer.

24 h before data acquisition, all participants underwent a familiarization session simulating the condition of the main experiment composed of one trial with five strokes at high intensity to become accustomed to swimming with the testing device. Before the experiment, participants performed a 45 min standardized warm-up on land and in water. The warm-up on land included arm swings in various positions (20 reps); walkout with twist (10 reps); elastic band pull-apart (20 reps); scapula pushups (10 reps); 1 kg med ball throws (5 reps); and squat jumps (5 reps). The warmup in water covered a total volume of 500 m and consisted of the following parts: 100 m swim (easy pace); 2 × 100 m swim (kick/drill); 4 × 25 m (12.5 m 90% of the 50 m race pace followed by 12.5 m easy); and 100 m easy swim. Participants also had 10 min of seated rest following the warm-up, in accordance with a previous study (Olstad et al., 2020). The main experimental procedure consisted of two trials of 25 m front crawl semi-tethered swimming with maximal effort (during a 1-day test session). To predict the maximum velocity, the number of trials should be minimized in order to avoid fatigue (Gonjo et al., 2020). The rest time between the trials was 6 min to achieve total recovery (Hancock et al., 2015). Previous studies have proven that in the depleted muscle 100% of the ATP and PCr are restored within 3–5 min after an “all-out” bout (Bogdanis et al., 1998). Furthermore, in the study by Danek et al. (2020) peak power output (PPO) during six repeated 10 s “all-out” bouts on the cycle ergometer, separated by 4-min of active recovery did not decrease. These results confirm the fact that a 6-min recovery was sufficient to restore the phosphagen source and allow the subject to perform maximal effort in consecutive trials. All trials were performed with training clothes preferred by the swimmers.

An in-water start was used in a prone position on the surface with the participant’s legs held by a coach’s assistant close to the wall. Participants began to swim at the sound of a whistle (without pushing off the wall). The swimmers were instructed to attain maximal swimming velocity as quickly as possible and to keep it for as long as possible. Furthermore, they were verbally encouraged before and during the test to maintain maximal effort. A previous study suggested that breathing patterns did not influence performance in tethered swimming (Amaro et al., 2017). Hence, the participants applied a free-breathing pattern for the front crawl technique. Referring to the relationship between short-term exercise with maximal intensity and blood lactate concentration values (Avlonitou, 1996), the applied sprint performance test with semi-tethered swimming was ensured to be anaerobic.

For all tests, the resisted load was set at the minimum (1 kg) while maintaining the cable tension. According to Cuenca-Fernández et al. (2020), a relatively low load (2.3 kg corresponded to 15% maximal load) during semi-tethered swimming allowed to obtain a maximal swimming velocity (1.97 m·s^-1^) which in this study indirectly indicated a potential to perform the maximal anaerobic effort. Furthermore, during trials, a swimming velocity would be close to a free-swimming condition. Setting a low load also had a benefit in minimizing the injury risk, as heavier loads could exceed their physical abilities and lead to injuries in adolescent swimmers (Pyne, 2021). Velocity data were recorded automatically for 15 m (between 3 m and 18 m). The starting point at 3 m was motivated by participant’s safety, to avoid the machine from pulling the swimmers back into the wall.

### Data analysis

**FIGURE 2:**
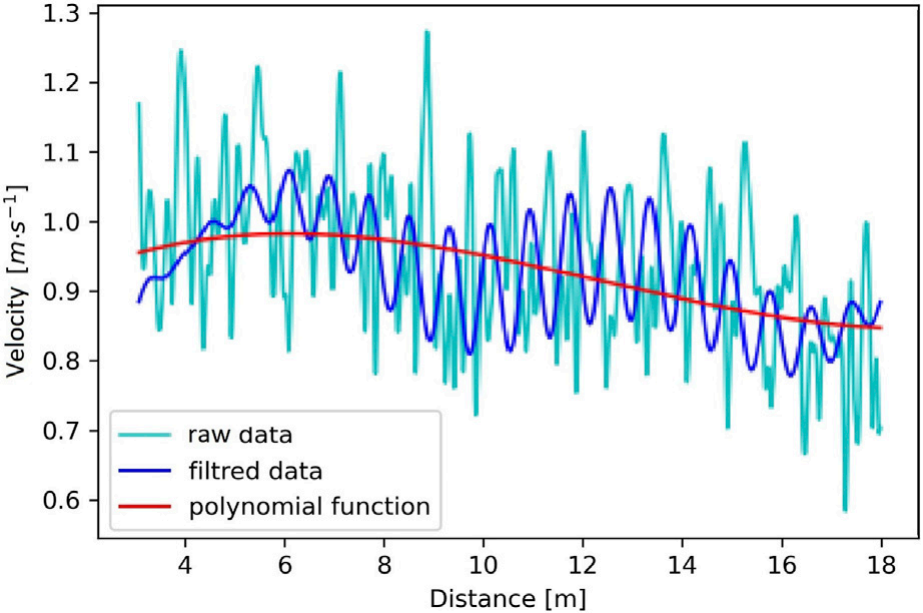
The raw data chart of registered individual instantaneous swimming velocity (acceleration and deceleration) and the chart of instantaneous swimming velocity in time/distance function after filtering using the FFT algorithm and the individual velocity profile based on the third-degree polynomial generated from the filtered data (study participant #2, trial #2).

The raw data was exported as instantaneous velocities in the form of a function of time/distance, obtained during the semi-tethered front crawl swimming for each participant (Figure 2). Data analysis was performed in the Python version 3.9.7 software package (Python Software Foundation, Wilmington, United States) and filtered using the Fast Fourier Transform (FFT) with a band-pass filter which only allows frequencies within a specific range determined by the lower and upper cutoff frequencies from 10 Hz to 85 Hz. (Crenna et al., 2021) (Figure 2). Those cutoff frequencies were chosen to keep the shape of the curve similar to the WAnT and to obtain detailed swimming velocity profile variables. The sample size of n = 8 was set with minimum acceptable reliability (ICC) (*ρ*_0_): 0.7, with the level of significance set as a = 0.05, and a power of 0.31 (Walter et al., 1998).

### Variables estimation

Subsequently, the optimal degree of the polynomial (third-degree polynomial) was selected from the filtered data series using the Akaike Information Criterion (AIC). AIC implements a model’s maximal likelihood estimation (log-likelihood) as a measure of its property of fit (Bozdogan, 1987). An individual velocity profile was estimated for each participant (where: the *X*-axis is time/distance; the *Y*-axis is the fitted polynomial) (Figure 2).

**FIGURE 3:**
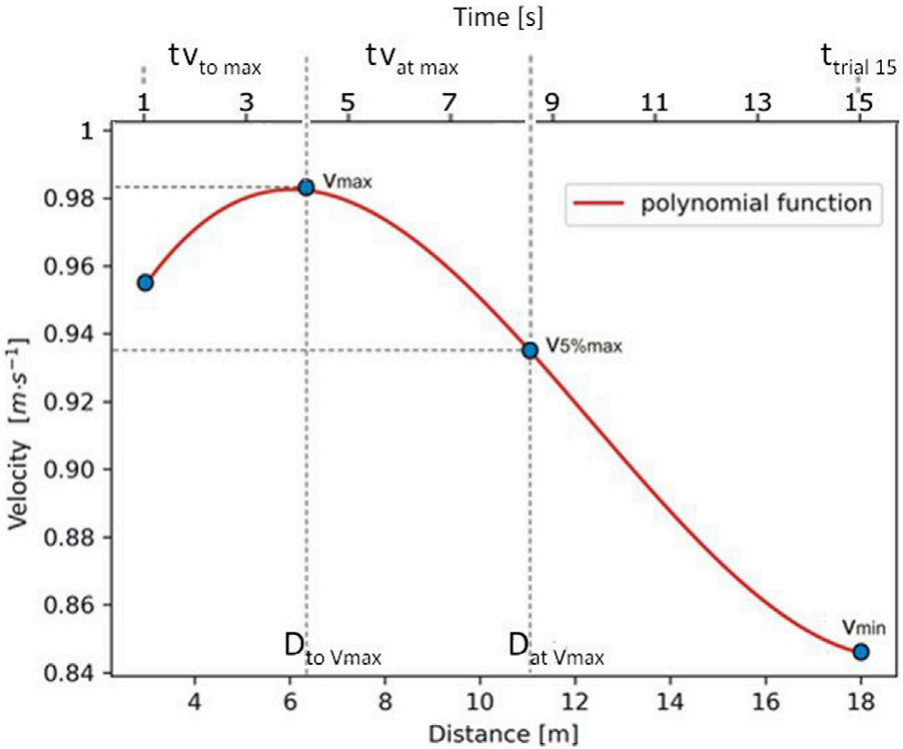
An individual instantaneous swimming velocity (acceleration and deceleration) profile in time/distance function, based on a third-degree polynomial generated from filtered data by marking the variables of anaerobic performance of an adolescent swimmer (study participant #2, trial #2). Note: v_max_ - maximal swimming velocity; v_min_ - minimal swimming velocity; D_to_ v_max_ - distance covered when v_max_ was reached; D_at_ v_max_ - distance covered while v_max_ was kept; tv_to_ _max_ - time to reach v_max_; tv_at_ _max_ - time interval between the point where v_max_ was reached and the point where v_max_ decreased by 5%; t_trial15_ - time to reach 15 m (from 3 m to 18 m).

From the polynomial velocity profile, time to reach 15 m (from 3 m to 18 m) (t_trial15_ [s]), maximal swimming velocity (v_max_ [m·s^-1^]), and minimal swimming velocity selected after reaching v_max_ for 18 m (v_min_ [m·s^-1^]) were obtained. Furthermore, detailed swimming velocity profile variables (Smolka and Ochmann, 2013): time to reach v_max_ (tv_to max_ [s]), time to maintain maximal swimming velocity (tv_at max_ [s]) defined as the time interval between the point where v_max_ is reached and the point where v_max_ decreased by 5%, distance covered when v_max_ was reached (D_to_ v_max_ [m]), distance covered while keeping v_max_ (D_at_ v_max_ [m]) (Figure 3). Additionally, the fatigue index (FI [%]) was calculated using the following formula (2) (Inbar et al., 1996):

$$\mathrm{FI}=\left(\left[v_{\max}\;‐v_{\min}\;\right]\cdot 100\right)\cdot v_{\max}^{‐1}$$ (2)

**FIGURE 4:**
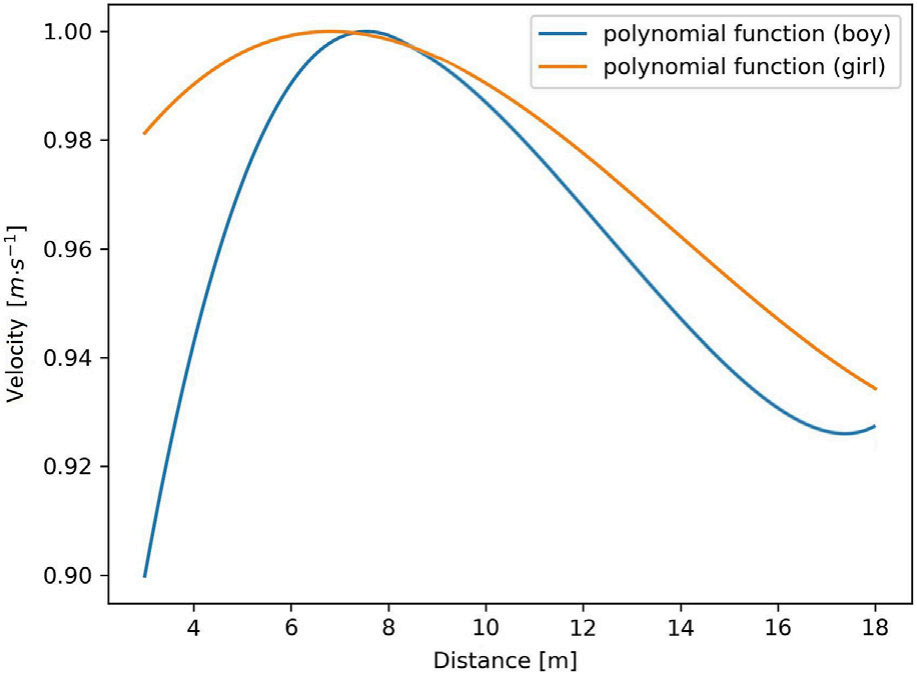
An example of normalized data with an individual velocity profile based on a polynomial curve (girl: study participant #2, trial #2; boy: study participant #1, trial #2).

Filtered data was normalized (scaled) in relation to the maximum velocity obtained during the semi-tethered swimming trials. Velocity data series was divided by v_max_ (range from v_min_·v_max_^−1^ to 1 was obtained, where 1 was the highest velocity). In regard to maximal abilities, a normalization of data on the vertical axis enabled objective comparison and evaluation of an individual’s instantaneous swimming velocity profile in the same scale for selected swimmers (Figure 4).

Each analyzed parameter was checked for relative and absolute reliability. Reliability is defined as the extent to which measurements can be replicated and reflects a degree of correlation and agreement between measurements (Motheral, 1998). The 95% confidence interval (95% CI), which defines the range within which the actual value of the statistic is 95% likely to fall, were determined for all reliability indicators.

The coefficient of variation (CV) (%) as an absolute reliability indicator was used to determine the similarity of the assessed variables within two measurements of one subject (intra-subject). This coefficient, as the ratio of the standard deviation to the mean, indicated the range of differentiation of a given parameter (variable dispersion). CV was interpreted as: CV<5% low variance; CV > 0.05 (or CV>5%) high variance (Hopkins et al., 2001). CV was calculated using the following formula (3):

$$\mathrm{CV}=\left(\mathrm{SD}\cdot M^{‐1}\right)\cdot 100$$ (3)

where: SD is the intra-individual standard deviation of both trials; M is the intra-individual mean of both trials).

The intraclass correlation coefficient (ICC) as a relative reliability indicator was a measure of the agreement between the results of the two trials. The relative reliability was classified as poor (ICC<0.50), moderate (0.50<ICC<0.69), good (0.70<ICC<0.89), or excellent (ICC≥0.90). A high ICC close to 1 indicated high similarity between values from the same assessed variables and *vice versa*. A low ICC close to zero means that values from the same variables are not similar (Trevethan, 2017). ICC with a two-way random, absolute agreement, single-measure model (Koo and Li, 2016) was calculated according to the following formula (4):

$$\mathrm{ICC}=\left({\mathrm{MS}}_{R}‐{\mathrm{MS}}_{E}\right)\cdot {\left({\mathrm{MS}}_{R}+\left(k‐1\right){\mathrm{MS}}_{E}\right)}^{\;‐1}+k\cdot n^{‐1}\left({\mathrm{MS}}_{C}‐{\mathrm{MS}}_{E}\right)$$ (4)

where: MS_R_ = mean square across rows of a matrix, MS_E_ = mean square error, MS_C_ = mean square across columns of a matrix, k = number of raters/measurements, and n = number of subjects.

Standard error of the mean (SEM) as an absolute reliability indicator has been defined as a determination of the amount of variation spread in the measurement’s error of the test (Harvill, 1991). A high SEM shows that sample means are widely spread around the mean (Weir and William, 2020). SEM was calculated according to the following formula (5):

$$\text{SEM}=\text{SD}\cdot \sqrt{\left(1\right.}‐\text{ICC})$$ (5)

where: SD is the inter-individual standard deviation of both trials.

Minimal detectable change (MDC) is an absolute reliability indicator of the minimal amount of change in the parameter that must occur in an individual to ensure that the change in score is not the effect of measurement error. MDC was used to differentiate between real change and random measurement error (Stratford and Riddle, 2012). MDC was calculated according to the following formula (6):

$$\text{MDC}=SEM\cdot 1.96\cdot \sqrt{2}$$ (6)

where: SEM−standard error of the mean.

**FIGURE 5:**
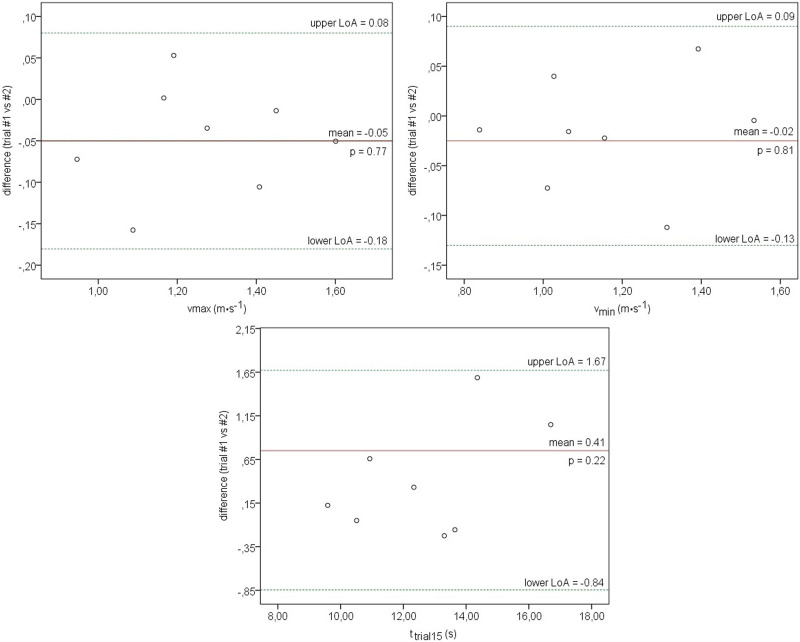
The Bland-Altman plot used to define limits of agreement between two measurements (trial #1 and #2) in the test with semi-tethered swimming for vmax, vmin, and ttrial15. The measure differences (*y*-axis) are delineated as a two-measure mean function (*x*-axis) at analyzed variables. The horizontal solid line represents the mean difference between the two measures. The two horizontal dotted lines represent the upper and the lower limit of agreement (1.96∙SD).

Bland-Altman plots were constructed to display agreement between two measurements (trials #1 and #2) in the analyzed variables. Limits of agreement (LoA) were used to compare individual differences between trial #1 and #2, where upper limit = mean difference+(SD·1.96) and lower limit = mean difference-(SD·1.96). Mean differences ±1.96 SD were provided for LoA lines. The *p* < 0.05 level was considered statistically significant (Bland and Altman, 1999) (Figure 5).

## Results

Figure 3 shows an individual velocity profile based on a polynomial curve from which the variables describe the potential to perform the maximal anaerobic effort of adolescent swimmers can be read: maximal velocity (v_max_), minimum velocity (v_min_), the distance (D_to_ v_max_) and time (tv_to max_) a swimmer need to reach maximal velocity and the distance (D_at_ v_max_) and time (tv_at max_) at which a swimmer can maintain maximal swimming velocity according to the criterion of a 5% decrease of v_max_, and time to reach 15 m (t_trial15_).

Figure 4 illustrates an example (one representative of a girl and a boy) of normalized data (range from v_min_·v_max_^−1^ to 1) with an individual velocity profile based on a third-degree polynomial curve.

Table 1 shows the relative and absolute reliability indicators (variability estimates (CV), relative reliability (ICC), and absolute reliability (SEM, MDC) for parameters plotting the velocity profile (v_max_, v_min_ and t_trial15_) and the variables describing this profile (tv_to max_, tv_at max_, D_to_ v_max_, D_at_ v_max_ and FI).

**TABLE 1:** Descriptive statistics of parameters plotting the individual velocity profile and variables describing this profile, with estimators of variability (CV), relative reliability (ICC), and absolute reliability (SEM, MDC).

	Trails_total_			Trail_#1_		Trail_#2_		Indexes of reliability			
Variables	M	SD	CI95%	M	SD	M	SD	CV	ICC	SEM	MDC
v_max_ (m·s^-1^)	1.27	0.21	1.16–1.37	1.24	0.22	1.29	0.21	3.63	0.88	0.07	0.20
v_min_ (m·s^-1^)	1.17	0.22	1.06–1.28	1.16	0.23	1.18	0.23	2.63	0.95	0.05	0.14
t_trial15_ (s)	12.67	2.27	11.54–13.80	12.88	2.49	12.46	2.18	2.71	0.98	0.35	0.97
tv_to max_ (s)	3.22	0.67	2.89–3.55	3.54	0.82	2.91	0.24	16.44	0.19	0.73	2.01
tv_at max_ (s)	5.04	1.73	4.18–5.90	5.51	2.27	4.57	0.86	18.17	0.01	1.72	4.76
D_to_ v_max_ (m)	4.13	1.08	3.59–4.66	4.59	1.32	3.66	0.52	16.47	0.50	0.76	2.12
D_at_ v_max_ (m)	6.07	2.01	5.07–7.07	6.41	2.55	5.73	1.37	17.68	0.24	1.76	4.87
FI (%)	8.12	3.77	6.24–9.99	6.98	4.15	9.25	3.21	39.89	0.48	2.73	7.56

Note: Variables obtained after fitting the polynomial to the series of filtered output data. Parameters plotting the individual velocity profile v_max_—maximal swimming velocity; v_min_—minimal swimming velocity; t_trial15_—trial time at 15 m. The variables describing the velocity profile tv_to max_—time to reach v_max_; tv_at max_—time at v_max_; D_to_ v_max_—distance covered when v_max_ was reached; D_at_ v_max_—distance covered while keeping v_max_; FI—fatigue index. M—mean, SD—standard deviation, CI95%—95% confidence interval, CV—coefficient of variation, ICC—intraclass correlation coefficient, SEM—standard error of the mean, MDC—minimal detectable change.

For the parameters plotting the individual velocity profile, CV showed low variance for v_max_, v_min_ and t_trial15_ (CV<5%). ICC_2,1_ for v_max_, v_min_ and trial t_trial15,_ demonstrated respectively good (ICC_2,1_: 0.88) very good (ICC_2,1_: 0.95), and excellent (ICC_2,1_: 0.98) rate of relative reliability. The absolute reliability indicators, i.e., SEM and MDC were higher for v_max_ (SEM: 0.07 m·s^-1^; MDC: 0.20 m·s^-1^) than v_min_ (SEM: 0.05 m·s^-1^; MDC: 0.14 m·s^-1^). However, in the t_trial15_ SEM was 0.35 s, while MDC 0.97 s.

The variables describing the velocity profile (Table 1) showed: high variance of CV for tv_to max_, tv_at max_, D_to_ v_max_ and D_at_ v_max_. ICC_2,1_ was moderate for D_to_ v_max_ (ICC_2,1_: 0.50) and weak for tv_to max_, tv_at max_, and D_at_ v_max_. Results concerning the absolute reliability indicators showed that tv_at max_ (SEM: 1.72 s; MDC: 4.76 s) was higher than tv_to max_ (SEM: 0.73 s; MDC: 2.01 s). D_at_ v_max_ (SEM: 1.76 m; MDC: 4.87 m) was higher than D_to_ v_max_ (SEM: 0.76 m; MDC: 2.12 m). The relative and absolute reliability indicators for fatigue index (FI) showed high variance (CV>5%), low rate of relative reliability (ICC: 0.48), and SEM and MDC indicators for FI were 2.73% and 7.56%, respectively.

Bland-Altman index revealed an acceptable agreement (LoA ≤5%) with no significant differences between the two measurements (trial #1 and #2) in the semi-tethered swimming test for vmax (*p* = 0.77), vmin (*p* = 0.81), and t_trial15_ (*p* = 0.22) (Figure 5).

## Discussion

In this study, the reliability of a front crawl sprint performance test based on the estimation of the individual instantaneous swimming velocity profiles in semi-tethered swimming was assessed. Among the analyzed variables, v_max_, v_min_, and t_trial15_ had reliable outcomes indicating low variability (CV<5%) and excellent relative reliability (ICC_2,1_ from 0.88 to 0.98) and absolute reliability (SEM from 0.07 to 0.35 and MDC from 0.14 to 0.97). Also, the Bland-Altman analysis showed an acceptable agreement (LoA ≤5%) with no significant differences between trial #1 and #2 in the test with semi-tethered swimming for v_max_ (*p* = 0.77), v_min_ (*p* = 0.81), and t_trial15_ (*p* = 0.22) (Figure 5). It can be assumed that v_max_, v_min,_ and t_trial15_ of the velocity profiles qualify them as objective indirect measures of an adolescent swimmer’s current potential to perform maximal anaerobic effort. This is partly consistent with Soares et al. (2014) where maximal swimming velocity (v_max_) is considered an indirect anaerobic performance metrics over short distances (50 m). Furthermore, Smolka and Ochmann (2013) reported that v_max_ and v_min_ were determinants of the sprint time in 100 m swimming (r = −0.90, r = −0.92, respectively).

When interpreting these results, attention should be made on how to obtain the v_max_ and improve the v_min_. A reflection of these adaptations is the maintenance of the fatigue index (FI) level or its reduction, which may increase swimming performance at sprint distances. In addition, the variables describing the velocity profile, tv_to max_, tv_at max_, D_to_ v_max_, D_at_ v_max_, and also FI were characterized by high variability (CV>5%) and slightly worse reliability in the relative dimensions (ICC from 0.01 to 0.50) and absolute SEM (0.73–2.73), and MDC (2.01–7.56). The high variability and low reliability in the following variables (tv_to max_, tv_at max_, D_to_ v_max_, D_at_ v_max_, FI) could be caused by starting the velocity measurement at a point located 3 m from the pool wall, where the study participants obtained different swimming velocities.

In the results from Demarie et al. (2019), strong correlations were found between the average power obtained in WAnT on a cycle ergometer and the average velocity obtained in the swimming test with IMU sensors for 75 m freestyle in a short (r = 0.809) and long course pool (r = 0.700). Additionally, the best times achieved by swimmers in the 50 m and 100 m freestyle races strongly correlated with the average swimming velocity obtained in the 75 m test (r = 0.659–0.952) and the average power in WAnT (r = 0.736–0.855) (Demarie et al., 2019). However, Dotan (2019) referred to the results of McKie et al. (2018) and suggested that the confrontation of the results of anaerobic tests carried out in a specific environment and under laboratory conditions (WAnT) is inappropriate and methodologically incorrect from the point of view of movement modality. Therefore, there are premises to believe that the semi-tethered swimming test enables keeping the natural water conditions, a similar muscular activity (Bollens et al., 1988), swimming stroke, and physiological responses to free swimming (Morouço et al., 2014; Ruiz-Navarro et al., 2020).

The adolescent swimmers in the current study performed their tests within the time characteristic of anaerobic tests (12.67 ± 2.27 s for swimming 15 m) (Table 1). Similarly, short tests lasting 10–15 s were used in the studies by Yeater et al. (1981) and Costill et al. (1986), with fully tethered swimming to assess the individual maximal power values representative of the ATP-PCr catabolism rate. Thus, a shorter test distance to assess the anaerobic potential opens the possibility of obtaining a higher maximal velocity (Inbar et al., 1976; Zajac et al., 1999). In this context, considering the psychological factors of effort maximization (Hatfiel and Landers, 1987; Zajac et al., 1999), the current argument seems to confirm the alternative application of the semi-tethered swimming test in water instead of using the Wingate test.

It seems that the basis for the lower t_trial15_ and v_max_ values (Table 1) is the shorter test duration time presented in the individual velocity profiles (polynomial curve) (Figures 2, 3). The discussed difference may also be due to too short of a rest interval between the first and second trial. The duration was adopted based on research conducted on adult swimmers (Hancock et al., 2015), which draws attention to the potentially greater susceptibility of adolescent swimmers to fatigue reactions (Bailey et al., 1995). However, it is presumed that 70% of the ATP and PCr are restored within 30 s, 90% of the rest value reaches within 2 min, and 100% within 3–5 min after an “all-out” effort (Hultman et al., 1967). Based on this suggestion, the low values of t_trial15_ and v_max_ (Table 1) obtained by the tested swimmers may result from the fact that due to their age and inexperience, they build their exercise capacity along with the elimination of deficits in the quality of their swimming technique (Lätt et al., 2009). In general, it should be assumed that the explanation of the problem discussed here requires further research, considering to extend both the water test duration and recovery time between performance trials.

Energetic contribution to a sprint-swim performance over 22 s in adults has been estimated, i.e.,: 38% from ATP-PCr system, 48% from glycolytic system, and 4% from the aerobic system (Rodríguez and Mader, 2021), with a greater contribution of the first two systems. As performance times decrease to 25 m, there is a larger aerobic contribution for longer performance times (slower swimmers, i.e., adolescents). Moreover, during the prepubertal stage, the anaerobic contribution to short-distance swims is likely to be lower compared to pubertal individuals (Mezzaroba and Machado, 2014). Hence, field-based anaerobic tests should be complemented with additional laboratory-based measurements, e.g., maximal accumulated oxygen deficit (Dekerle, 2021). In the past, anaerobic potential to produce high-power output was measured during maximal 30 s cycling and arm cranking tests among 13-year-old boys and girls (Hawley et al., 1992) and adolescent swimmers aged 16 (Strzala and Tyka, 2009). A correlation of around r = 0.650 between high-power output and swimming performance <100 m distances suggests sprint performance test (to 50 m) may be appropriate to evaluate the anaerobic potential of adolescent swimmers. The field-based semi-tethered test used in this study enables assessment of the current state of performing maximal anaerobic effort in sprint swimming through individual velocity profiles. This approach allows for monitoring the adaptive changes during the training process at any given time and is critical in the context of improving training routines. This leads to the belief that the results obtained from the created velocity profiles during semi-tethered swimming may be the basis for modifying the quantitative and qualitative components of the training load to increase the exercise capacity of adolescent swimmers in sprint races. The practical value of this study indicates that using the field-based semi-tethered test, a coach can compare two swimmers with the same trial time and different trajectories of velocity profiles. Furthermore, the simplicity of such tests encourages their use in swimming profiling.

### Limitations of the study

The current study has some potential limitations. The first potential limitation refers to the significant relationships between the average swimming velocity and the average power obtained in WAnT on a cycle ergometer (Demarie et al., 2019). In this context, the concept that the classic WAnT assesses the ability to perform anaerobic performance while cycling is inadequate for assessment in specific conditions of water exercise. This was also adopted by Dotan (2006). A second potential limitation is the small number of study participants (eight), which may limit the decisive dimension of the formulated conclusions. Nevertheless, the observed results create conditions for extending research toward a larger group of swimmers. A small research sample (twelve swimmers) was used by Smolka and Ochmann (2013). Thirdly, it is worth noting that girls present a maturation process earlier than boys (being in the same age range), and their different metabolic profiles may affect velocity abilities. The use of data normalization allows comparisons among different swimmers on the same scale (Figure 4). A small sample without division by gender was used by Zamparo et al. (2000) (nine teenage swimmers). Moreover, the individual exercise capacity of swimmers in the scope of their preferred swimming techniques, and distance should be taken into consideration. Fourthly, the 1 kg load was the same for every participant but represented a different relative load for each of them. Considering the effect of different loads on semi-tethered swimming and its relationship with the power curves, 1 kg may not have been enough to generate maximum power. Cuenca-Fernández et al. (2020), found that among 18 years old competitive male swimmers on semi-tethered swimming, the load eliciting the peak power (71.38 W) was 6.00 kg which was 45% maximal load and corresponded to a swimming velocity of 0.92 m·s^-1^. In the present study, it was not the PPO, but maximal swimming velocity was one of the indicators of a potential to perform the maximal anaerobic effort (Driss and Vandewalle, 2013). Referring to Cuenca-Fernández et al. (2020), the lowest external load (2.3 kg) gave the possibility to reach a maximal swimming velocity (1.97 m·s^-1^). Another potential limitation is how the beginning of the test was conducted. Perhaps pushing off the wall would be more representative of the competition condition because the swimmers after the start and turn have acceleration above zero. Furthermore, it should be also taken into account that the resistance offered by the added mass may be higher underwater given the quadratic relationship of the hydrodynamic drag (Marinho et al., 2009). Hence, the external work was higher because of the increases in the load and the drag caused by the load when accelerating (Dominguez-Castells et al., 2013). A relatively long trial time would also be desirable. Finally, the mean limitation of the 1080 Sprint was examined by Bergkvist et al. (2015) under a dry-land condition. However, the swimming environment could produce specific issues, such as swimmers potentially kicking the cable or the waves affecting the movements of the cable, which would affect the velocity data. Hence, there is applying on-land accuracy data in-water conditions. Furthermore, to minimize errors, the device was placed on top of the starting block so that the only part of the cable attaching to the swimmer’s body was the end of it.

Future research should provide more information about these potential limitations of the current research. Nevertheless, this study contributes to the level of knowledge available in the literature about adolescent swimmers. This study can be an instruction manual for those who want to study the topic with a greater number of subjects, in other populations, or in different conditions.

## Conclusion

To conclude, the changes in instantaneous swimming velocity in time/distance during front crawl semi-tethered sprint test with maximal intensity can be analyzed as individual mathematical models of velocity profiles in adolescent swimmers. The findings revealed that the v_max_, v_min_ and t_trial15_ estimated from these profiles were considered reliable and can be assigned specificity characteristics. Consequently, the applied procedure allows objective and individualized quantification of the selected parameters to indirectly evaluate the current level of adolescent swimmers’ potential to sustain maximal effort in the anaerobic condition. The observation of the swimming velocity profiles including reliable parameters allows for monitoring the adaptive changes in the current state of performing maximal anaerobic effort matters during the training process. As a result of a field-based semi-tethered test, the coach may identify the capacity for improvement and provide guidelines to athletes for the preparation of specific training sessions for adolescent swimmers. The results obtained from the created velocity profiles may occur point of reference for modifying the quantitative and qualitative components of the training load in order to enhance the exercise capacity of adolescent swimmers in sprint performance. Future studies should further explore these aspects to provide coaches and athletes with more detailed and valuable information to better inform training decisions.

## Data Availability

The datasets presented in this article are not readily available because of concerns over the risk of inadvertent disclosure of young athletes’ personal health information and performance information. Requests to access the datasets should be directed to stefan.szczepan@awf.wroc.pl.
